# Immunopathogenesis of severe pneumonia in children with emphasis on CD4+ T cells, Tim-3 and cytokine-mediated immune dysregulation

**DOI:** 10.3389/fmed.2026.1884262

**Published:** 2026-07-16

**Authors:** Fang Cao, Qi Zhang, Lunan Yan

**Affiliations:** Department of Clinical Laboratory, Jinhua Maternal and Child Health Care Hospital, Jinhua, Zhejiang, China

**Keywords:** biomarker, CD4+ T cells, children, cytokines, HAVCR2, IL-1β, IL-6, IL-8

## Abstract

Severe pneumonia in children remains a major cause of hospitalization, respiratory failure and infection-related mortality. Increasing evidence indicates that disease severity is shaped not only by pathogen burden but also by the timing, intensity and compartmentalization of the host immune response. CD4+ T cells are central to this process because they coordinate macrophage activation, B-cell help, mucosal defense and immune resolution. In severe disease, however, CD4+ T-cell responses may become depleted, misdirected or constrained by immune-checkpoint pathways. T-cell immunoglobulin and mucin-domain-containing protein 3 (Tim-3/HAVCR2) is a context-dependent immune regulator that may reflect activation, adaptive restraint, regulatory compensation or exhaustion-like dysfunction depending on disease phase and inflammatory milieu. In parallel, cytokines such as interleukin (IL)-6, IL-8, IL-1β and tumor necrosis factor-α amplify neutrophilic inflammation, endothelial activation, vascular leakage and systemic injury. This narrative review synthesizes current evidence on CD4+ T-cell dysfunction, Tim-3 biology and cytokine-mediated immune dysregulation in severe pediatric pneumonia. We propose a checkpointed hyperinflammation framework in which innate cytokine amplification coexists with constrained adaptive immune coordination. However, direct pediatric pneumonia evidence remains limited, and much of the mechanistic interpretation of Tim-3 is extrapolated from adult sepsis, oncologic, chronic infection and autoimmune literature. CD4+Tim-3, soluble Tim-3, and focused cytokine panels therefore can be evaluated in longitudinal pediatric cohorts with pathogen stratification, multi-checkpoint profiling, and blood–airway immune comparison.

## 1 Introduction

Pneumonia remains one of the most consequential infectious syndromes in childhood, not only because of its global burden but also because of its capacity to progress rapidly from a localized respiratory infection to life-threatening systemic disease (1). Despite substantial advances in childhood vaccination, antimicrobial treatment, oxygen delivery, pulse-oximetry screening, and pediatric intensive care, severe pneumonia continues to cause major morbidity and mortality, particularly among children younger than 5 years and in health-care settings where delayed diagnosis, limited critical-care capacity, malnutrition, or incomplete immunization increase vulnerability (2–4). However, severe pneumonia is not a homogeneous disease, children with the same pathogen may have very different outcomes; for instance, some will respond rapidly to supportive care and pathogen-specific antimicrobials, while others will develop persistent fever, hypoxemia, respiratory failure, shock, or multiple organ dysfunction (5–7). This heterogeneity implies that pathogen invasion is not the sole determinant of disease severity. Rather, the intensity, timing and control of the immune response are now known to play a key role in disease outcome (8).

Traditionally, pediatric pneumonia has been viewed from a pathogen-focused perspective, with a focus on bacterial, viral, atypical, or mixed pathogens (1). While this is critical for therapeutic purposes, it only partially reflects the biology of severe disease (1, 9). The lung is a dynamic immunological milieu in which airway epithelial cells, alveolar macrophages, dendritic cells, neutrophils, monocytes, endothelial cells and lymphocytes communicate via antigen presentation, chemokine gradients, cytokines, metabolic stress responses and pathways of tissue damage (10). When appropriately regulated, inflammation supports pathogen clearance and tissue repair. When it is excessive or not well regulated, these pathways can lead to alveolar-capillary leak, mucus plugging, neutrophil-induced damage, gas-exchange dysfunction, and systemic inflammation (11). In children, these processes are also influenced by developmental immunity. The immune system of children is not a scaled-down version of the adult immune system, but has age-related patterns of innate activation, adaptive memory, thresholds for regulatory feedback, and vulnerability to inflammatory damage (12, 13). Within this complex immune network, CD4+ T cells occupy a particularly important position. They coordinate macrophage activation, B-cell help, cytotoxic T-cell support, mucosal defense, and immune resolution through functionally diverse subsets, including Th1, Th17, T follicular helper, and regulatory T cells (14, 15). In severe pneumonia, however, CD4+ T-cell responses may become quantitatively reduced, phenotypically skewed, or functionally constrained. Such changes can weaken pathogen clearance while allowing innate inflammation to persist (16, 17). T-cell immunoglobulin and mucin-domain-containing protein 3 (Tim-3) has therefore emerged as a biologically relevant checkpoint molecule in this setting. Although frequently described as an inhibitory receptor, Tim-3 is better understood as a context-dependent immune regulator whose meaning varies according to cell type, ligand availability, antigen persistence, disease phase, and cytokine milieu (18, 19). Tim-3 expression on CD4+ T cells may therefore reflect activation, adaptive braking, exhaustion-like dysfunction, or regulatory compensation rather than a single fixed immune state (20).

Despite increasing interest in immune biomarkers for pediatric pneumonia, important knowledge gaps remain. Most clinical studies evaluate inflammatory markers such as C-reactive protein, procalcitonin, interleukin-6 (IL-6), or interleukin-8 (IL-8) without integrating adaptive immune status. Conversely, studies of lymphocyte subsets often lack sufficient cytokine context or longitudinal sampling (21). As a result, the mechanistic relationship among CD4+ T-cell dysfunction, Tim-3 signaling, and cytokine-mediated injury remains incompletely defined. This review synthesizes current evidence on the immunopathogenesis of severe pneumonia in children, with particular emphasis on CD4+ T cells, Tim-3, and key cytokine networks. We first outline the immune architecture of severe pediatric pneumonia, then discuss CD4+ T-cell remodeling and Tim-3 biology and finally evaluate how cytokine and checkpoint biomarkers may support risk stratification and future immune-guided research.

## 2 Review methodology

This article was designed as a narrative review using a structured literature-search strategy. It was not conducted as a systematic review or meta-analysis, and no formal quantitative synthesis, risk-of-bias assessment or protocol registration was performed. The purpose was to synthesize mechanistic and translational evidence relevant to severe pediatric pneumonia, with particular emphasis on CD4+ T-cell dysfunction, Tim-3 biology, immune-checkpoint pathways and cytokine-mediated immune dysregulation. Literature searches were conducted in PubMed/MEDLINE, Scopus and Web of Science, with additional targeted searches in Google Scholar. The search covered publications from January 2000 to June 2026, while earlier landmark articles were included when they provided foundational information on Tim-3 biology, T-cell exhaustion, pediatric pneumonia classification or immune-checkpoint regulation.

Search terms combined pediatric pneumonia, severe disease, T-cell immunity, Tim-3/HAVCR2, immune checkpoints and cytokine-mediated inflammation. Eligible studies included clinical, cohort, case-control, translational, mechanistic and review articles addressing severe pneumonia or lower respiratory tract infection in children; CD4+ T-cell responses, lymphocyte subsets, checkpoint markers or cytokine profiles in pneumonia, respiratory infection, sepsis or related inflammatory conditions; and clinically relevant evidence on biomarkers, severity stratification, respiratory failure, intensive-care admission or outcomes. Studies were excluded if they were unrelated to pneumonia, respiratory infection, T-cell immunity, cytokine biology or immune checkpoints; lacked sufficient methodological detail; were unavailable in full text; or were not published in English. Animal and *in vitro* studies were included selectively when mechanistically relevant. Because this was a narrative review, evidence was synthesized thematically around immune dysregulation in severe pediatric pneumonia, CD4+ T-cell dysfunction, Tim-3 and related checkpoint pathways, and cytokine-mediated hyperinflammation.

## 3 Immune dysregulation at the center of severe pediatric pneumonia

### 3.1 The developing lung as an immunological battlefield: from protective inflammation to tissue injury

Severe pediatric pneumonia is increasingly understood as a syndrome of infection-driven immune dysregulation rather than pathogen invasion alone. Although microbial exposure initiates disease, clinical outcome depends on how the developing immune system senses, amplifies, contains and resolves inflammation (22). This is particularly important in infants and young children because pediatric immunity is not simply a weaker version of adult immunity. Age-related differences in innate activation, dendritic-cell maturation, adaptive memory and regulatory thresholds mean that the same pathogen can trigger different immune programs across children (23, 24). A short-lived protective cytokine response in one child may therefore become prolonged, uncontrolled and tissue-damaging in another.

The airway epithelium is the first point of contact between pathogens and host immunity. Infected epithelial cells act as sentinel cells by detecting microbial and damage-associated signals and releasing chemokines, alarmins and cytokines (25). These mediators recruit neutrophils, monocytes, dendritic cells and lymphocytes into the lung, shaping the early inflammatory environment. When this response is coordinated, innate cells restrict pathogen growth, antigen-presenting cells support adaptive immunity and regulatory pathways promote repair (26, 27). In severe pneumonia, however, this balance is lost. IL-1β and TNF-α amplify fever, endothelial activation, vascular adhesion and myeloid recruitment; IL-6 promotes acute-phase signaling, vascular permeability and systemic inflammation; and IL-8 drives neutrophil influx, protease release, reactive oxygen species production and neutrophil extracellular trap formation (28–30). These processes are protective when transient but pathogenic when sustained.

This feed-forward inflammatory cycle helps explain why severe pneumonia may present with hypoxemia, respiratory failure, circulatory dysfunction and systemic inflammatory markers before microbiological results are available (31). Conventional markers such as C-reactive protein and procalcitonin remain clinically useful, but they provide limited insight into the immune trajectory. They do not explain why some children show both persistent inflammatory activation and impaired adaptive immune coordination (32). Therefore, lymphocyte phenotyping, cytokine profiling and immune-checkpoint assessment may provide additional mechanistic and prognostic information in severe pediatric pneumonia.

### 3.2 CD4+ T cells as coordinators of pulmonary immunity

CD4+ T cells play a central regulatory role in this inflammatory network, extending beyond conventional helper functions. CD4+ T cells orchestrate macrophage activation, assist B-cell differentiation and antibody quality, direct cytotoxic T-cell responses and contribute to the resolution or persistence of lung inflammation (33–35). Th1 cells activate macrophage antimicrobial activity and control of intracellular pathogens; T follicular helper cells support humoral immunity; regulatory T cells prevent excessive tissue damage; and Th17 cells enhance mucosal immunity and recruit neutrophils to fight extracellular pathogens (36, 37).

But the same CD4+ T-cell responses that benefit the host can also cause disease when dysregulated. Th17-mediated inflammation, for instance, may be beneficial for mucosal immunity but can exacerbate neutrophil-driven lung injury (38). This is the case with childhood Mycoplasma pneumoniae pneumonia. Children with the disease have pathogen-specific IFN-γ-producing CD4+ effector-memory T cells that appear to contribute to disease (39). However, increased Th17 responses have been linked to severe or resistant disease, implying that CD4+ T-cell responses can transition from protective coordination to inflammatory amplification in the absence of regulatory control (40, 41).

### 3.3 Dysfunctional CD4+ T-cell states in severe disease

CD4+ T-cell dysfunction in severe pneumonia may take several forms. The first is quantitative depletion or redistribution, in which peripheral CD4+ T cells are lost while immune responses are amplified in the lung or lymph nodes. The second is qualitative misdirection, in which helper T-cell differentiation is excessive, unstable or mismatched to the pathogen (42, 43). This includes excessive Th17 or insufficient regulatory activity. The third is checkpoint-constrained dysfunction, in which CD4+ T cells are present but are limited in their ability to proliferate, produce balanced cytokines, and provide effective help (43, 44).

These conditions are not mutually exclusive. A child with severe pneumonia may have systemic lymphopenia, intense airway inflammation, and dysfunctional T cells. This is clinically relevant because it challenges a binary distinction between hyperinflammation and immunosuppression. Both processes may coexist in severe pneumonia. This perspective has been reinforced by single-cell and immune-census studies in pneumonia, which have shown exhausted or dysfunction-associated CD4+ memory subsets in severe lung infection (45, 46). Recent bronchoalveolar single-cell studies in pediatric Mycoplasma pneumoniae pneumonia also indicate that peripheral blood may not be the only source of information about the immune response, as airway immune cells can show more pronounced inflammatory and differentiation signatures than peripheral cells (47). Therefore, circulating biomarkers and lymphocyte counts can be regarded as convenient but limited surrogates for the lung immune environment.

CD4+ T-cell dysfunction in severe pneumonia is likely to occur across a spectrum rather than as a single abnormal state. As summarized in Table 1, these dysfunctional patterns include quantitative depletion or redistribution, qualitative helper T-cell misdirection, checkpoint-constrained dysfunction, and combined inflammatory-checkpoint states.

**Table 1:** Major dysfunctional CD4+ T-cell states in severe pediatric pneumonia.

Dysfunction phenotype	Description	Possible immune readout	Interpretation	Potential clinical implication
Quantitative depletion or redistribution	Peripheral CD4+ T cells decrease while immune activity may persist in lung tissue or lymphoid compartments.	Low circulating CD4+ count or percentage.	Peripheral blood may underestimate tissue-level immune activation.	May indicate reduced adaptive immune reserve, risk of delayed recovery, or need for serial lymphocyte monitoring rather than relying on a single blood measurement.
Qualitative misdirection	CD4+ differentiation is excessive, unstable or mismatched to pathogen type.	Skewed Th17, Th1, Treg or effector-memory signatures.	CD4+ response may amplify inflammation rather than coordinate recovery.	Can identify children with immune-mediated lung injury, resistant inflammation, or pathogen-specific immune patterns requiring closer clinical follow-up.
Checkpoint-constrained dysfunction	CD4+ T cells are present but functionally restrained by checkpoint pathways.	Increased CD4+Tim-3, possibly with PD-1, CTLA-4, LAG-3 or TIGIT.	Suggests adaptive immune braking, regulatory compensation or exhaustion-like dysfunction.	May indicate impaired helper T-cell coordination, delayed pathogen clearance, prolonged respiratory support, or need for multi-checkpoint immune profiling.
Combined inflammatory-checkpoint state	High cytokines coexist with impaired adaptive immune coordination.	High IL-6/IL-8 plus high CD4+Tim-3 or sTim-3.	Fits the proposed checkpointed hyperinflammation model.	May identify a higher-risk inflammatory-checkpoint phenotype associated with persistent inflammation, delayed resolution, intensive-care requirement or prolonged hospitalization.

This table summarizes the principal patterns of CD4+ T-cell dysfunction described in severe pediatric pneumonia, including quantitative depletion or redistribution, qualitative helper T-cell misdirection, checkpoint-constrained dysfunction, and combined inflammatory-checkpoint states. Together, these phenotypes illustrate how CD4+ T-cell abnormalities may contribute to persistent inflammation, impaired immune coordination, and failure of immune resolution.

### 3.4 Tim-3 as a checkpoint for activation, suppression and exhaustion

T-cell immunoglobulin and mucin-domain-containing protein 3 (Tim-3), encoded by HAVCR2, may provide a mechanistic link between CD4+ T-cell activation and dysfunction, although this interpretation in severe pediatric pneumonia remains partly extrapolated from other disease settings. Originally identified as an inhibitory molecule in Th1 responses, Tim-3 is now known as a context-specific immune regulator expressed on CD4+ and CD8+ T cells, regulatory T cells, natural killer cells, macrophages, and dendritic cells (48, 49). Its significance varies with time, cell type, ligand presence, antigen persistence, and the cytokine milieu. Tim-3 may be a marker of recent activation in some contexts, adaptive braking to prevent tissue damage in others, and an exhaustion-like state with diminished effector function in cells that have been persistently stimulated (50).

These features make Tim-3 a relevant candidate marker for investigation in severe pediatric pneumonia. The inflamed lung is rich in many of the signals that trigger checkpoint remodeling, including sustained antigen exposure, elevated cytokine levels, hypoxia, metabolic stress, and T-cell receptor stimulation (50, 51). Tim-3 has multiple ligands and binding partners, including galectin-9, CEACAM1, HMGB1 and phosphatidylserine (52). In T cells, Tim-3 signaling is regulated by factors such as BAT3. BAT3 dissociation can lead to downstream signaling that promotes inhibitory pathways that limit T-cell receptor signaling, proliferation and cytokine production (53). In dendritic cells, Tim-3-HMGB1 interactions may regulate nucleic-acid sensing and innate immune responses (54).

Therefore, Tim-3 here serve as broader immune-checkpoint network rather than as an isolated receptor. Because PD-1, CTLA-4, LAG-3 and TIGIT regulate T-cell activation through partly distinct mechanisms, their comparison is necessary to clarify what Tim-3 can and cannot indicate in severe pediatric pneumonia (55–57). The major alternative checkpoint pathways are discussed below.

**Figure 1:**
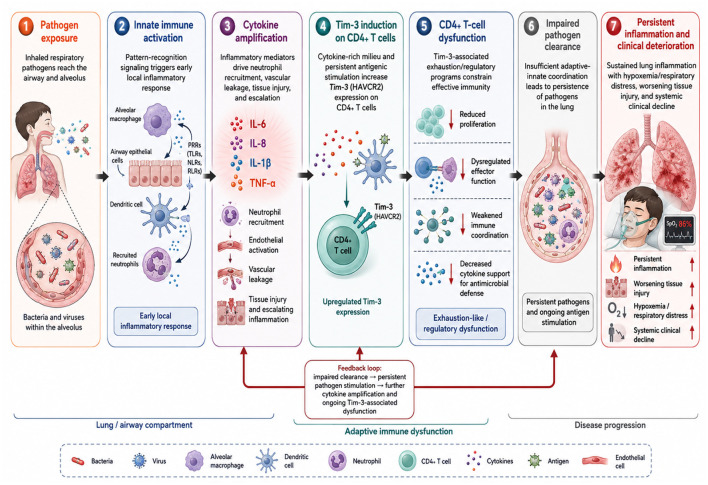
Proposed mechanistic model of immune dysregulation in severe pediatric pneumonia. This schematic illustrates a pathway linking pathogen exposure, innate immune activation, cytokine amplification, Tim-3 induction on CD4+ T cells, CD4+ T-cell dysfunction, impaired pathogen clearance, persistent inflammation and clinical deterioration. Inhaled bacterial, viral or atypical pathogens activate airway epithelial cells, alveolar macrophages, dendritic cells and recruited neutrophils, promoting cytokine amplification through IL-6, IL-8, IL-1β and TNF-α. Persistent antigenic stimulation and cytokine-rich inflammation may induce Tim-3/HAVCR2 expression on CD4+ T cells, contributing to reduced proliferation, dysregulated effector function and weakened immune coordination. Impaired pathogen clearance may then sustain antigen exposure and cytokine amplification, creating a feed-forward loop that promotes persistent lung inflammation, hypoxemia, tissue injury and clinical deterioration. This model is conceptual and requires validation in longitudinal pediatric pneumonia cohorts. Created with BioRender.

These findings, derived largely from non-pediatric pneumonia settings, suggest that Tim-3 is not simply a marker of exhausted T cells but may function as a broader regulatory node linking adaptive T-cell responses, innate immune sensing and inflammation control. Figure 1 presents a stepwise model showing how pathogen exposure may initiate innate immune activation, cytokine amplification, Tim-3 induction on CD4+ T cells, CD4+ T-cell dysfunction, impaired pathogen clearance, persistent inflammation and clinical deterioration in severe pediatric pneumonia. The figure is intended as a conceptual summary of Tim-3-associated mechanisms rather than direct proof of a validated pediatric pneumonia pathway.

### 3.5 Evidence provenance and translational limits of Tim-3 literature

Because direct pediatric pneumonia data on CD4+Tim-3 remain limited, the Tim-3 literature discussed in this review is best considered according to its source of evidence. Evidence directly relevant to pediatric pneumonia currently supports only selected components of the proposed framework, including severe pneumonia-associated cytokine elevation, lymphocyte alterations, compartmentalized blood–airway inflammation, and the reported prognostic value of soluble Tim-3 in children with severe pneumonia (16, 58–60). In contrast, many mechanistic details of Tim-3 biology, including ligand-dependent signaling, co-expression with other inhibitory receptors, exhaustion-associated transcriptional programs, and checkpoint-mediated immune restraint, are derived mainly from cancer immunology, chronic viral infection, autoimmunity and sepsis studies(48–52).

This distinction is important because these disease contexts are not interchangeable with acute severe pneumonia in children. Cancer and chronic viral infection typically involve prolonged antigen exposure, persistent immune stimulation and long-term remodeling of T-cell states, whereas pediatric pneumonia is usually acute, pathogen-diverse and influenced by developmental immunity, vaccination status, nutritional state, timing of antimicrobial therapy, airway compartmentalization and age-specific inflammatory thresholds (12, 13, 61). Adult sepsis studies are also informative but may not fully reflect pediatric pulmonary infection, because systemic sepsis-related immune paralysis differs from localized or compartmentalized lung inflammation in children (13, 61). Therefore, Tim-3 expression in severe pediatric pneumonia may cautiously act as a marker of Tim-3-associated immune remodeling rather than direct evidence of T-cell exhaustion, immune paralysis or causal checkpoint-mediated pathology (62).

Accordingly, conclusions drawn from Tim-3 studies in cancer, chronic infection or sepsis are used here to support mechanistic plausibility, not to claim definitive pediatric pneumonia mechanisms (62). Direct validation will require pediatric cohorts that simultaneously measure CD4+Tim-3, soluble Tim-3, PD-1, CTLA-4, LAG-3, TIGIT, CD4+ T-cell functional readouts and cytokine profiles across disease phases (13, 61). Until such studies are available, Tim-3 can be described as a candidate biomarker and maybe checkpoint marker in severe pediatric pneumonia, rather than as an established driver of disease severity.

### 3.6 Comparative immune-checkpoint pathways beyond Tim-3

Although Tim-3 is the focus of this review, it is presented here as a part of a broader immune-checkpoint network rather than as an isolated marker. CD4+ T-cell dysfunction in severe inflammatory disease may involve several co-inhibitory receptors, including PD-1, CTLA-4, LAG-3 and TIGIT. These pathways regulate T-cell activation, proliferation, cytokine production and immune restraint through different mechanisms, and each may provide distinct information about the timing and functional state of immune regulation in severe pediatric pneumonia (55–57, 62, 63).

PD-1 is commonly linked with persistent antigen exposure and reduced T-cell effector activity, although in acute severe pneumonia it may also reflect recent activation or adaptive restraint rather than true exhaustion. CTLA-4 mainly regulates early costimulatory signaling by competing with CD28 for CD80/CD86 binding and is closely related to regulatory T-cell activity. LAG-3 may indicate deeper checkpoint co-expression and a more constrained CD4+ T-cell phenotype, especially when expressed with Tim-3 or PD-1. TIGIT may further contribute to broader suppression of T-cell and natural killer cell responses. However, direct pediatric pneumonia data for CTLA-4, LAG-3 and TIGIT remain limited (55, 56, 62).

Overall, comparison with these alternative checkpoint pathways helps clarify why Tim-3 is biologically important but insufficient as a standalone biomarker. Future studies should evaluate Tim-3 together with PD-1, CTLA-4, LAG-3 and TIGIT, alongside CD4+ T-cell counts, functional assays, cytokine profiles and longitudinal clinical outcomes. This broader approach may distinguish transient immune activation from regulatory compensation, checkpoint-associated dysfunction and exhaustion-like immune states in severe pediatric pneumonia.

### 3.7 Cytokine-mediated immune dysregulation

Cytokine-mediated inflammation is a central feature of severe pediatric pneumonia, but the key cytokines do not act in the same way. IL-6, IL-8, IL-1β and TNF-α are most frequently implicated, while IL-10 and IFN-γ provide important regulatory and immune-contextual information (64–66). IL-6 connects local lung inflammation with systemic responses such as fever, acute-phase activation, vascular leak and metabolic stress (67, 68). IL-8 is more closely linked to pulmonary neutrophil recruitment, mucus plugging and alveolar injury, particularly in children with severe hypoxemic disease (69, 70).

IL-1β and TNF-α function as upstream inflammatory amplifiers. They promote myeloid activation, endothelial dysfunction, vascular adhesion and tissue injury, helping convert a localized infection into a broader inflammatory lung-damage process (71, 72). IL-10 has a more complex role. It may initially help restrain excessive inflammation, but persistent IL-10 elevation, especially when accompanied by high Tim-3 expression, may suggest failed immune resolution or an immune-paralysis-like state (73). Therefore, cytokines should not be interpreted as isolated markers; they are better understood as part of a dynamic inflammatory-regulatory network.

**Figure 2:**
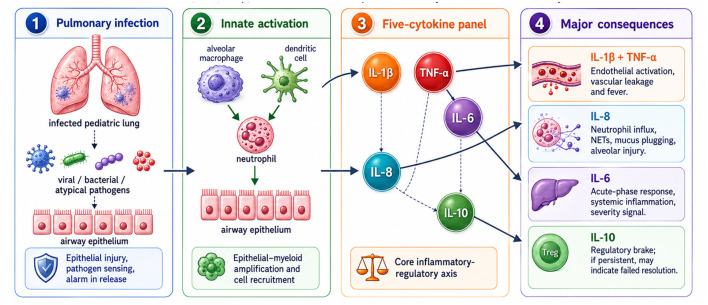
Simplified cytokine network in severe pediatric pneumonia. Pulmonary infection by viral, bacterial, atypical, or mixed pathogens activates airway epithelial cells and innate immune cells, including alveolar macrophages, dendritic cells, and neutrophils. This epithelial–myeloid activation promotes a focused cytokine network involving IL-1β, TNF-α, IL-6, IL-8, and IL-10. IL-1β and TNF-α function as upstream inflammatory amplifiers that promote endothelial activation, fever, vascular leakage, and tissue injury. IL-8 primarily drives neutrophil recruitment, neutrophil extracellular trap formation, mucus plugging, and alveolar damage. IL-6 links local pulmonary inflammation with systemic acute-phase responses and clinical severity. IL-10 represents a compensatory regulatory signal that may limit excessive inflammation, although persistent IL-10 elevation in severe disease may indicate failed immune resolution. This simplified schematic emphasizes the core inflammatory–regulatory cytokine axis most relevant to severe pediatric pneumonia and supports interpretation of cytokine profiles alongside CD4+ T-cell status, Tim-3 expression, pathogen type, sampling compartment, and clinical trajectory. Created with BioRender.

Pediatric data support this network-based interpretation. High IL-6 and IL-8 have been associated with severe pneumonia requiring mechanical ventilation, while studies in Mycoplasma pneumoniae pneumonia show that cytokine patterns vary with disease severity (74, 75). Airway-focused studies further suggest that local pulmonary immune responses may reflect lung injury more accurately than serum cytokines alone (76). As summarized in Figure 2, cytokine-mediated dysregulation in pediatric pneumonia involves interconnected pathways rather than single linear mediators, supporting a systems-level interpretation of disease severity and the need to assess cytokine profiles alongside CD4+ T-cell status, Tim-3 expression, pathogen type, sampling compartment and clinical trajectory.

## 4 Checkpointed hyperinflammation: a translational framework for severe pediatric pneumonia

Herein, we use the term checkpointed hyperinflammation as a translational framework rather than as a confirmed pediatric pneumonia mechanism. The framework is proposed to describe a possible immune configuration in which epithelial–myeloid cytokine amplification coexists with constrained adaptive CD4+ T-cell coordination. This interpretation is mechanistically relevant because severe pneumonia can involve simultaneous inflammatory amplification, lymphocyte redistribution, altered helper T-cell function and increased checkpoint-marker expression. However, direct pediatric evidence linking CD4+Tim-3 expression, soluble Tim-3 and cytokine dynamics within the same severe pneumonia cohorts remains limited. Therefore, checkpointed hyperinflammation should be regarded as a testable model that integrates existing observations, not as a proven endotype.

### 4.1 Conceptual basis and distinction from immune exhaustion

Two partial paradigms are commonly used to describe severe pediatric pneumonia. One emphasizes hyperinflammation, in which excessive cytokine production drives lung injury and respiratory failure. The other emphasizes immune suppression or exhaustion, in which dysfunctional lymphocytes fail to clear pathogens and prolong disease (61). In practice, these processes may occur together. A child with severe pneumonia may show high inflammatory cytokines, fever, hypoxemia and neutrophil-dominated lung injury, while also showing lymphopenia, CD4+ T-cell dysfunction or increased checkpoint-marker expression (77). This suggests that severe disease is not simply a cytokine storm or a purely exhausted immune state, but a hybrid immune configuration.

In this review, checkpointed hyperinflammation describes this specific, testable immune configuration. The model links two interacting processes: persistent epithelial–myeloid cytokine amplification and progressive adaptive immune constraint. In the first process, airway epithelial cells, macrophages, monocytes, dendritic cells and infiltrating neutrophils release cytokines such as IL-6, IL-8, IL-1β and TNF-α, promoting endothelial activation, neutrophil influx, vascular leak and lung injury (78, 79). In the second process, persistent antigen exposure, hypoxia, metabolic stress and inflammatory cytokines may alter CD4+ T-cell function, leading to depletion, tissue sequestration, pathogenic helper-cell skewing or checkpoint-mediated restraint (80). Tim-3 may sit at the intersection of these processes because its expression on CD4+ T cells can reflect activation, adaptive restraint, regulatory compensation or exhaustion-like dysfunction depending on disease phase and inflammatory context (81).

This framework is not intended to rename immune dysregulation or immune exhaustion. Immune dysregulation is a broad term for maladaptive immune responses, while immune exhaustion usually refers more specifically to persistent antigen-driven T-cell dysfunction with reduced proliferation, impaired effector cytokine production and increased inhibitory receptor expression (62). Checkpointed hyperinflammation is more specific because it is compartmentally integrative, temporally dynamic and translationally testable. It links innate cytokine amplification with CD4+ T-cell checkpoint remodeling, interprets Tim-3 according to inflammatory context, and predicts that children with concurrent high cytokines and high CD4+Tim-3 or soluble Tim-3 may represent a distinct inflammatory-checkpoint phenotype (55). This distinction may help explain why some children have persistent inflammation, delayed pathogen clearance and clinical deterioration despite antimicrobial and supportive therapy.

### 4.2 Evidence status of the checkpointed hyperinflammation model

The checkpointed hyperinflammation model should be interpreted as a framework rather than a confirmed pediatric pneumonia endotype. Current evidence supports several components of the model: severe pediatric pneumonia is associated with increased inflammatory cytokines such as IL-6, IL-8, IL-1β and TNF-α; CD4+ T-cell number, distribution or function may change during severe infection; and soluble Tim-3 has been reported as a potential prognostic marker in children with severe pneumonia. Mechanistic studies from other disease settings also support the biological plausibility that Tim-3 may reflect activation, immune restraint, regulatory compensation or exhaustion-like T-cell states.

These observations may converge in some children as a combined inflammatory-checkpoint state, in which cytokine amplification remains active while adaptive CD4+ T-cell coordination becomes constrained by checkpoint-associated remodeling. However, direct pediatric validation remains limited. Future studies can test whether cytokine elevation, CD4+Tim-3 expression, soluble Tim-3 and broader checkpoint-marker expression occur together in the same patients, whether this combined profile predicts severity or delayed recovery, and whether longitudinal Tim-3 changes distinguish transient activation from persistent checkpoint-associated dysfunction.

### 4.3 Translational implications and biomarker applications

Checkpointed hyperinflammation provides a way to interpret children who show both strong inflammatory activation and signs of adaptive immune dysfunction. Elevated IL-6 or IL-8 may indicate ongoing cytokine-driven inflammation, whereas increased CD4+Tim-3 expression or soluble Tim-3 may suggest that the CD4+ T-cell compartment is under regulatory or exhaustion-like pressure. For this reason, a single biomarker is unlikely to capture the full immune state. A cytokine-only approach may identify inflammation but miss adaptive immune restraint, while a Tim-3-only approach may overlook the inflammatory context in which Tim-3 is expressed. A more informative strategy may combine CD4+Tim-3 expression, soluble Tim-3 and a focused cytokine panel including IL-6, IL-8, IL-1β, TNF-α and IL-10, although this requires prospective validation (82, 83).

This integrated approach may also support risk stratification and disease monitoring. For example, a child with high IL-6 and IL-8 but preserved CD4+ T-cell reserve may represent a cytokine-predominant inflammatory phenotype. In contrast, a child with similar cytokine elevation but high CD4+Tim-3, low CD4+ T-cell reserve or rising soluble Tim-3 may represent a combined inflammatory-checkpoint phenotype (84). These children may appear similar at admission but may differ in pathogen clearance, oxygen requirement, risk of secondary infection, need for intensive care, and recovery trajectory (85). Because severe pneumonia changes over time, serial immune measurements are likely to be more useful than a single admission sample (86). Importantly, these biomarkers can be viewed as adjuncts to clinical assessment, not replacements for history, examination, imaging, microbiology, oxygen support and conventional inflammatory markers (87).

**Figure 3:**
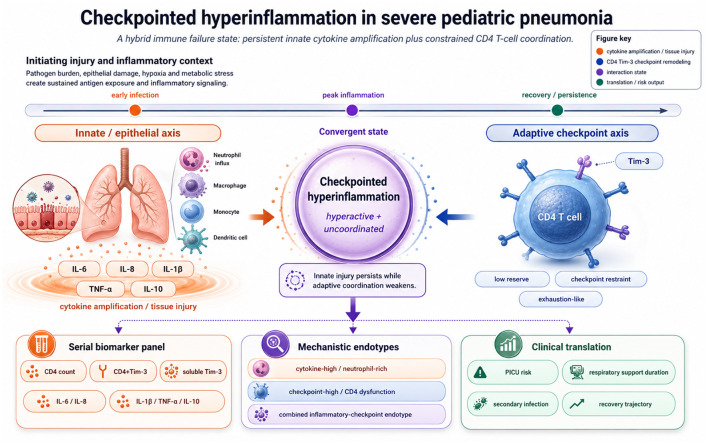
The figure illustrates a hybrid immune state in which sustained epithelial and innate immune activation drives cytokine amplification, neutrophil influx, endothelial activation and lung injury, while persistent antigen exposure and inflammatory stress promote CD4+ T-cell dysfunction and Tim-3 checkpoint remodeling. Together, these processes are proposed to define “checkpointed hyperinflammation,” a immune configuration in which inflammation remains active while adaptive immune coordination may be constrained. Serial profiling of CD4+ T-cell reserve, CD4+Tim-3 expression, soluble Tim-3 and cytokines may help test whether inflammatory, checkpoint-high and combined immune endotypes exist in severe pediatric pneumonia. Created with BioRender.

Translationally, this framework supports both biomarker development and mechanistic phenotyping. A practical panel could include CD4+ T-cell count or percentage, CD4+Tim-3 frequency or mean fluorescence intensity, soluble Tim-3 and selected cytokines such as IL-6, IL-8, IL-1β, TNF-α and IL-10 (88, 89). Such profiling can distinguish cytokine-high, checkpoint-high and combined inflammatory-checkpoint endotypes, and may eventually guide patient selection for trials of host-directed therapies. The central issue is not whether Tim-3 or cytokines are elevated alone, but whether Tim-3-high CD4+ T-cell states occur together with persistent cytokine activation and whether this combined signature predicts severity, treatment response or outcome (90). As summarized in Figure 3, checkpointed hyperinflammation is therefore proposed as a dynamic immune-failure model in which innate inflammatory injury and adaptive checkpoint remodeling co-evolve in severe pediatric pneumonia (59).

## 5 Limitations and future validation requirements

Several limitations should be considered when interpreting the checkpointed hyperinflammation framework and the broader evidence summarized in this review.

### 5.1 Limited direct pediatric evidence for Tim-3 biology

The first limitation is the limited amount of direct pediatric pneumonia-specific evidence regarding Tim-3 biology. Existing pediatric studies support selected components of the proposed framework, including cytokine elevation, lymphocyte alterations, compartmentalized airway inflammation and the possible prognostic value of soluble Tim-3 (16, 58–60). However, few studies have simultaneously assessed CD4+Tim-3 expression, soluble Tim-3, Tim-3 ligand interactions, broader checkpoint pathways, cytokine profiles, CD4+ T-cell function and clinical outcomes in the same pediatric pneumonia cohorts. Tim-3 should currently be interpreted as a candidate biomarker and hypothesis-generating marker of immune-checkpoint remodeling rather than as an established causal driver of severe pediatric pneumonia (62).

### 5.2 Translational limits of extrapolated checkpoint literature

A second limitation is disease-context transferability. Much of the mechanistic understanding of Tim-3 signaling, ligand interactions, co-expression with other inhibitory receptors and exhaustion-like T-cell dysfunction comes from cancer immunology, chronic viral infection, autoimmunity and sepsis studies(48–52). These settings provide important biological insight but are not fully equivalent to acute severe pneumonia in children. Cancer and chronic viral infection usually involve prolonged antigen exposure and long-term immune remodeling, whereas pediatric pneumonia is often acute, pathogen-diverse and influenced by developmental immunity, vaccination status, nutritional state, timing of antimicrobial therapy, airway compartmentalization and age-specific inflammatory thresholds (12, 13, 61). Therefore, mechanistic conclusions from these fields should be applied cautiously, and Tim-3 expression in severe pediatric pneumonia should not be interpreted as direct proof of T-cell exhaustion, immune paralysis or causal checkpoint-mediated pathology (62).

### 5.3 Etiological and host heterogeneity in severe pediatric pneumonia

A third limitation is the heterogeneity of severe pediatric pneumonia itself. Severe pneumonia is a clinical syndrome rather than a single biological entity. Viral, bacterial, atypical and mixed infections may produce overlapping clinical manifestations but distinct immune programs (1, 9). Host factors such as age, nutritional status, vaccination history, previous antibiotic or corticosteroid exposure, comorbid disease and timing of sample collection may further influence cytokine and checkpoint profiles. Future studies should therefore stratify analyses by pathogen class, disease phase and clinically relevant host factors rather than treating severe pneumonia as a uniform immune phenotype (40, 41).

### 5.4 Peripheral blood vs. pulmonary immune compartments

**Figure 4:**
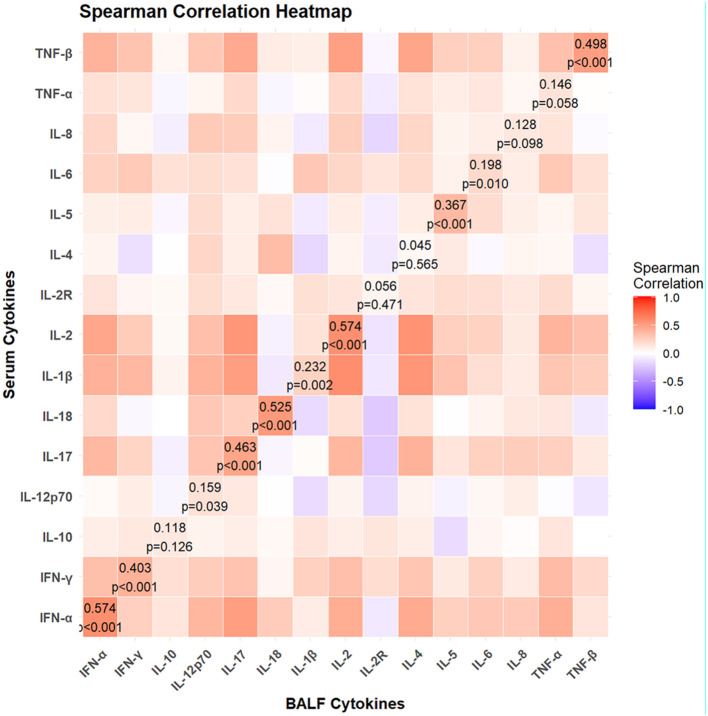
Spearman correlation heatmap showing relationships between local and systemic cytokines in children with lung consolidation; color intensity indicates correlation strength (red positive, blue negative), with significant correlations (*p* < 0.05) labeled with rs and p-values. Reproduced with permission from (61).

A fourth limitation is the difference between peripheral blood biomarkers and the pulmonary immune microenvironment. Blood sampling is practical and safer in children, but severe pneumonia is primarily localized in the lung, where airway epithelial cells, alveolar macrophages, dendritic cells, neutrophils and tissue-resident lymphocytes interact within a compartmentalized inflammatory niche. Peripheral CD4+ T-cell counts, CD4+Tim-3 expression and serum cytokines may therefore underestimate or misrepresent immune events occurring in bronchoalveolar or lung tissue compartments (90–92). When ethically and clinically feasible, future studies should compare blood-based immune profiles with airway samples such as bronchoalveolar lavage fluid, tracheal aspirates or other respiratory specimens obtained during routine care. This compartmental limitation is illustrated by Figure 4, which shows that local and systemic cytokine correlations in children with pneumonia-associated lung consolidation are heterogeneous and only partially overlapping. These findings support the need to interpret serum cytokines, CD4+Tim-3 expression and soluble Tim-3 as accessible but incomplete surrogates for the pulmonary immune microenvironment.

### 5.5 Need for longitudinal pediatric validation and future research priorities

The most important limitation of the current evidence base is the lack of longitudinal pediatric datasets that validate the complete checkpointed hyperinflammation model. Future research should move beyond isolated measurements of Tim-3, soluble Tim-3 or inflammatory cytokines and instead use integrated immune profiling across disease phases. Serial sampling during admission, peak inflammation and recovery is needed to determine whether Tim-3 expression reflects early activation, adaptive restraint, regulatory compensation, exhaustion-like dysfunction or persistent immune stress at different stages of severe pneumonia (62).

Future studies should also account for the clinical and biological heterogeneity of severe pediatric pneumonia. Analyses should be stratified by pathogen class, disease phase, age and relevant host factors rather than treating severe pneumonia as a single immune phenotype. Multi-checkpoint profiling should include Tim-3 together with PD-1, CTLA-4, LAG-3 and TIGIT, alongside CD4+ T-cell functional assays, soluble Tim-3 measurement and focused cytokine panels including IL-6, IL-8, IL-1β, TNF-α and IL-10 (55–57, 62, 63). Where ethically and clinically feasible, peripheral blood findings should also be compared with airway immune profiles because circulating biomarkers may not fully represent the pulmonary immune microenvironment (90–92).

A composite biomarker approach incorporating CD4+ T-cell status, CD4+Tim-3 expression, soluble Tim-3, broader checkpoint markers and cytokine profiles may help clarify whether checkpointed hyperinflammation represents a reproducible immune signature in a subset of children with severe pneumonia. Such studies should link immune trajectories with clinically meaningful outcomes, including hypoxemia, respiratory support, pediatric intensive-care admission, secondary infection, duration of hospitalization and recovery. Until these data are available, checkpointed hyperinflammation should be regarded as a testable conceptual framework rather than a confirmed pediatric pneumonia endotype.

## 6 Conclusion

Severe pediatric pneumonia is best understood as an infection-driven immune dysregulation syndrome in which pathogen burden interacts with developmental immunity, cytokine amplification and adaptive immune remodeling. CD4+ T cells are central to this process because they help determine whether pulmonary inflammation remains coordinated, becomes tissue-damaging or fails to support pathogen clearance. Within this context, Tim-3 is biologically important not as a standalone exhaustion marker, but as a context-dependent checkpoint molecule that may reflect activation, adaptive restraint, regulatory compensation or exhaustion like dysfunction depending on disease phase and inflammatory milieu.

This review proposes checkpointed hyperinflammation as a framework in which persistent innate cytokine activation coexists with constrained CD4+ T-cell coordination. A composite biomarker approach incorporating CD4+Tim-3 expression, soluble Tim-3 and focused cytokine panels may provide more meaningful information than conventional inflammatory markers alone. However, direct pediatric pneumonia evidence remains limited, and the model should not yet be interpreted as a confirmed disease endotype. Future longitudinal pediatric studies should validate whether Tim-3, broader checkpoint markers, cytokine profiles and CD4+ T-cell functional changes jointly predict severity, pathogen clearance, respiratory support, intensive-care admission and recovery. This approach could support more precise immune phenotyping and clinically useful biomarker development in severe pediatric pneumonia.
